# Updated evidence-based treatment of male sexual dysfunction among spinal cord injury patients

**Published:** 2012-11

**Authors:** Radin Maheronnaghsh, Ali Yousefian, Vafa Rahimi-Movaghar

**Affiliations:** ^a^Sina Trauma and Surgery Research Center, Tehran University of Medical Sciences, Tehran, Iran.; ^*b*^Research Centre for Neural Repair, University of Tehran, Tehran, Iran.

## Abstract

**Background:**

Decreased sexual function is a major concern of men with spinal cord injury (SCI). Erectile dysfunction (ED) and infertility can occur and affect men of any age with subsequent serious impacts on the quality of life. This paper was aimed to conduct a comprehensive review on the latest evidence-based works on prevention and treatment of ED and male infertility.

**Methods:**

The MedLine/PubMed was used as the data source to search and collect studies published during January 1, 1995 to August 1, 2012. Cross referencing of discovered articles was also performed. All of the evidences related to male sexual dysfunction in SCI patients were extracted. We designed search strategies using following keywords in both text word and subject heading forms: sexual dysfunction, erectile dysfunction, male infertility, spinal cord injury, veteran, evidence, prevention and treatment. According to the inclusion criteria, 83 studies were selected for further analyses.

**Results:**

There are three common methods proposed in the literature for ED treatment of including medication, surgical procedures, and vacuum constriction devices. Male infertility could be caused by ED, ejaculatory dysfunction and poor sperm quality. Assisted ejaculation and surgical sperm retrieval might be useful.

**Conclusions:**

Treatment of sexual dysfunction would decrease concern in spinal cord injured patients. Several medications and surgical treatments are now available to manage this problem in SCI population. In conclusion, regarding the importance of sexual satisfaction in SCI patients, considering different methods of sexual dysfunction management would lead to higher quality of life and better adherence to rehabilitation programs.

**Keywords:**

Sexual dysfunction, Erectile dysfunction, Male infertility, Spinal cord injury, Treatment


					Volume 4, Suppl. 1 Nov 2012
Publisher: Kermanshah University of Medical Sciences
URL: http://www.jivresearch.org
Copyright:
Congress of Iranian Neurosurgeons, 14-16 November 2012, Kermanshah, Iran
Paper No. 58
Updated evidence-based treatment of male sexual
dysfunction among spinal cord injury patients
Radin Maheronnaghsh a
, Ali Yousefian a
, Vafa Rahimi-Movaghar a,b,*
a
Sina Trauma and Surgery Research Center, Tehran University of Medical Sciences, Tehran, Iran.
b
Research Centre for Neural Repair, University of Tehran, Tehran, Iran.
Abstract:
Background: Decreased sexual function is a major concern of men with spinal cord injury (SCI). Erectile dysfunction
(ED) and infertility can occur and affect men of any age with subsequent serious impacts on the quality of life. This
paper was aimed to conduct a comprehensive review on the latest evidence-based works on prevention and
treatment of ED and male infertility.
Methods: The MedLine/PubMed was used as the data source to search and collect studies published during January
1, 1995 to August 1, 2012. Cross referencing of discovered articles was also performed. All of the evidences
related to male sexual dysfunction in SCI patients were extracted. We designed search strategies using following
keywords in both text word and subject heading forms: sexual dysfunction, erectile dysfunction, male infertility, spinal
cord injury, veteran, evidence, prevention and treatment. According to the inclusion criteria, 83 studies were selected
for further analyses.
Results: There are three common methods proposed in the literature for ED treatment of including medication,
surgical procedures, and vacuum constriction devices. Male infertility could be caused by ED, ejaculatory dysfunction
and poor sperm quality. Assisted ejaculation and surgical sperm retrieval might be useful.
Conclusions: Treatment of sexual dysfunction would decrease concern in spinal cord injured patients. Several
medications and surgical treatments are now available to manage this problem in SCI population. In conclusion,
regarding the importance of sexual satisfaction in SCI patients, considering different methods of sexual dysfunction
management would lead to higher quality of life and better adherence to rehabilitation programs.
Keywords:
Sexual dysfunction, Erectile dysfunction, Male infertility, Spinal cord injury, Treatment
* Corresponding Author at:
Vafa Rahimi-Movaghar: Associate professor of Neurosurgery, Research Deputy, Sina Trauma and Surgery Research Center, Sina Hospital, Hassan-
Abad Square, Imam Khomeini Ave, Tehran University of Medical Sciences, Tehran, Iran. Phone: (+98) 915 342 2682, (+98) 216 6757010,
Fax: (+98) 216 675 7009, Email: v_rahimi@sina.tums.ac.ir , v_rahimi@yahoo.com, (Rahimi Movaghar V.).

				

